# Neonatal Procedural Pain Disrupts Phosphorylation of KCC2 in the Spinal Cord

**DOI:** 10.1002/dneu.22993

**Published:** 2025-08-11

**Authors:** Mathilde Baudat, Elbert A. J Joosten, Sinno H. P. Simons, Daniël L. A. van den Hove, Renzo J. M. Riemens

**Affiliations:** ^1^ Department of Anesthesiology and Pain Management Maastricht University Medical Centre+ Maastricht the Netherlands; ^2^ Department of Translational Neuroscience, Mental Health and Neuroscience Research Institute Maastricht University Maastricht the Netherlands; ^3^ Department of Neonatal and Pediatric Intensive Care, Division of Neonatology Erasmus University Medical Centre Rotterdam‐Sophia Children Hospital Rotterdam the Netherlands; ^4^ Department of Psychiatry and Neuropsychology, Mental Health and Neuroscience Research Institute Maastricht University Maastricht the Netherlands

**Keywords:** GABAergic shift, epigenetic, neonatal pain, neurodevelopment, oxytocin

## Abstract

Neonatal procedural pain experienced in the neonatal intensive care unit can lead to long‐lasting remodeling of the central nervous system and, in particular, of the spinal nociceptive network. Preclinical studies indicate a disrupted inhibitory versus excitatory balance in the spinal cord due to reduced γ‐aminobutyric acid (GABA) ergic neurotransmission. During neonatal development a GABAergic shift occurs, which is regulated by the potassium‐chloride co‐transporter 2 (KCC2) and its oxytocin receptor (OXTR)‐dependent phosphorylation at the serine 940 residue (pKCC2). As DNA methylation of *Oxtr* is sensitive to early life adversity, such as neonatal procedural pain, we hypothesized that neonatal procedural pain reduces *Oxtr* methylation in the lumbar spinal cord and subsequently prevents the developmental increase in KCC2 and pKCC2. Using a rat model of repetitive neonatal procedural pain, four needle pricks were applied to the left hind paw every day from postnatal day (P)0 to P7. Spinal cord samples were collected at P0 and P10 to assess the levels of KCC2 and pKCC2 via Western blot analysis. Additionally, spinal *Oxtr* methylation was quantified using bisulfite pyrosequencing. The results indicated that neonatal procedural pain downregulates spinal pKCC2 levels, while KCC2 levels remain unchanged. These findings suggest a disrupted KCC2‐dependent chloride outflow and support the hypothesis that neonatal procedural pain disrupts the GABAergic shift. A developmental decrease in pKCC2/KCC2 levels was also observed in the ipsilateral spinal cord of P10 animals, indicating the involvement of other post‐translational mechanisms in the developmental regulation of spinal KCC2. Methylation of the *Oxtr* does not seem to be related to the disturbed GABAergic shift, given that no significant changes in *Oxtr* promoter methylation were detected. Overall, this study demonstrates that neonatal procedural pain disrupts spinal KCC2 phosphorylation and supports the hypothesis that neonatal procedural pain alters the GABAergic shift in the spinal cord.

## 1 Introduction

Preterm infants (< 37 weeks of pregnancy) are often admitted to the neonatal intensive care unit (NICU), where they receive an average of 10 to 14 painful procedures daily (Carbajal et al. 2008). In addition to the acute experience of pain, these painful procedures interfere with the activity‐dependent maturation of the central nervous system (CNS), with long‐lasting consequences on pain perception (Hathway et al. 2006; van den Hoogen et al. 2018; Li et al. 2015; Li and Baccei 2014). Current research on the effects of neonatal painful procedures hints at changes in the spinal nociceptive network and more specifically on γ‐aminobutyric acid (GABA) ergic inhibition (van den Hoogen et al. 2018; de Kort et al. 2022a; Li et al. 2015; Li and Baccei 2014).

**FIGURE 1:**
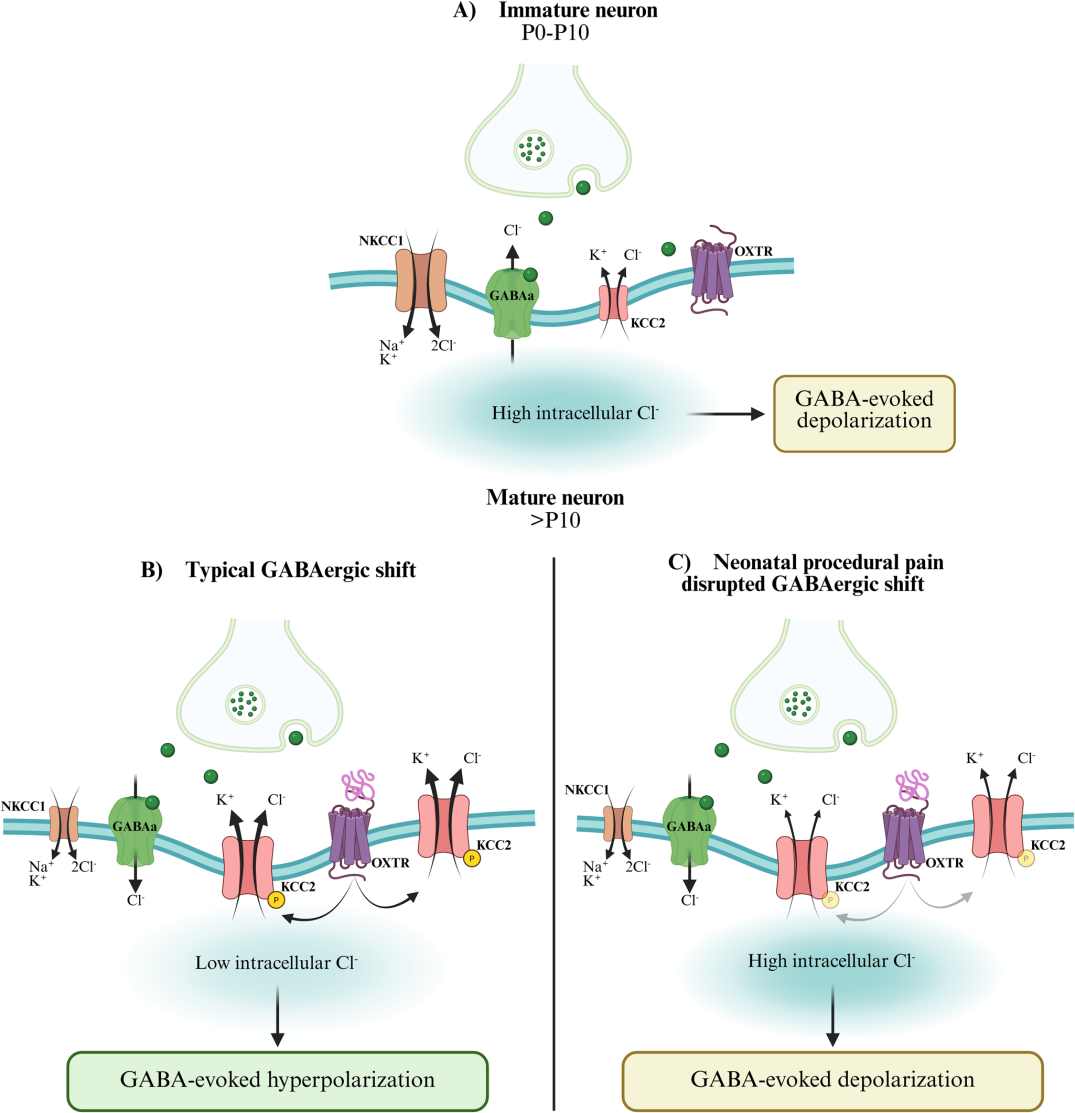
**The developmental GABAergic shift is triggered by OT‐OXTR interaction and neonatal procedural pain would disrupt the pathways**. (A) GABA_A_‐responding post‐synaptic neurons (blue) exhibit elevated levels of NKCC1 and low levels of KCC2. Consequently, the release of GABA by GABAergic interneurons (green) results in a high intracellular Cl^−^ concentration, causing GABA‐evoked depolarization in post‐synaptic neurons of the spinal cord. (B) In the typical development, the increase in KCC2 and pKCC2, enables lower intracellular Cl^−^ concentration and GABA‐evoked hyperpolarization of post‐synaptic neurons. Recent evidence from Leonzino and colleagues (Leonzino et al. 2016) demonstrates the essential role of OT binding to OXTR for promptly completing the GABAergic shift. In rodents, completion of the GABAergic shift occurs around P10. (C) Results from our study established that neonatal procedural pain diminished pKCC2 in the spinal cord, suggesting an impaired GABAergic shift, and subsequently, an enduring GABA‐evoked depolarization of the spinal cord. Created with BioRender.com.

In the first post‐natal week, GABAergic signaling goes through a highly conserved developmental shift, with immature neurons experiencing GABA‐induced depolarization due to elevated intracellular chloride (Cl^−^) levels (Figure 1A). As development progresses toward mature neurons, the expression of potassium‐chloride cotransporter 2 (KCC2) increases, lowering intracellular Cl^−^ concentrations and allowing GABA to mediate hyperpolarization and inhibition (Figure 1B) (Ben‐Ari 2002). This GABAergic process is fine‐tuned through the sodium‐potassium‐chloride co‐transporter (NKCC1), KCC2, and the phosphorylation of KCC2 at the serine 940 residue (Ser940) (Ben‐Ari 2002; Gazzo et al. 2021; Lee et al. 2011, 2007; Leonzino et al. 2016; McArdle et al. 2023; Stil et al. 2009; Watanabe et al. 2019).

Under healthy, typical maturation, the GABAergic shift and the increased KCC2 expression are established by P10 (Leonzino et al. 2016; Stil et al. 2009). Nevertheless, early‐life experiences like neonatal maternal separation are known to inhibit KCC2 expression in the hippocampus, whereas neonatal spinal cord injury downregulates KCC2 expression in spinal moto‐neurons in vitro, resulting in a loss of GABAergic inhibition (Furukawa et al. 2017; Gackiè and Vinay 2015). Furthermore, neonatal pain leads to enhanced firing of spinal projection neurons in adulthood, a negative membrane potential, and reduced spontaneous activity of spinal GABAergic neurons (van den Hoogen et al. 2020, 2018; Knaepen et al. 2013; Li et al. 2015; Li and Baccei 2014). The enhanced excitability of the spinal nociceptive network may be mediated by mechanisms of central sensitization, such as the recruitment of post‐synaptic N‐methyl‐D‐aspartate (NMDA) receptors and impaired GABAergic neurotransmission (Basbaum et al. 2009; van den Hoogen et al. 2018). Given the established vulnerability of the nociceptive network and more specifically of GABAergic neurotransmission to neonatal procedural pain, the present study aimed to determine whether neonatal procedural pain impairs GABAergic inhibition in the spinal dorsal horn by interfering with the developmental GABAergic shift. More specifically, we hypothesized that neonatal procedural pain disrupts KCC2 and its phosphorylation processes at the Ser940 residue (pKCC2), delaying or preventing the completion of the GABAergic shift.

Recent studies have highlighted that oxytocin (OXT) is a key regulator of the GABAergic shift (Leonzino et al. 2016; Tyzio et al. 2006). OXT, produced in the paraventricular nucleus (PVN), is critical for labor, social bonding, and modulation of nociception (Ben‐Ari et al. 2018; Poisbeau et al. 2018). OXT's role in pain regulation is linked to GABAergic inhibition in the spinal cord's lamina II via descending fibers from the PVN (Breton et al. 2008). Furthermore, OXT and the oxytocin receptor (OXTR) promote developmental KCC2 expression and phosphorylation at Ser940 (pKCC2) through the protein kinase C (PKC) pathway (Leonzino et al. 2016). Early‐life environmental stimuli, such as maternal care, influence *Oxtr* methylation, with evidence suggesting that *Oxtr* methylation may affect the GABAergic shift by modulating KCC2 expression (Bales et al. 2007; Kraaijenvanger et al. 2019; Leonzino et al. 2016; Unternaehrer et al. 2016). For instance, symptoms of maternal depression during the last trimester of pregnancy correlated with *OXTR* methylation in the newborn (Unternaehrer et al. 2016). Increased methylation of *Oxtr* and decreased OXTR protein expression were also encountered in prairie voles exposed to low levels of parental care (Bales et al. 2007). Lower levels of OXTR in the CNS have been shown to affect the GABAergic shift, as *Oxtr* knock‐out mice showed a decreased expression of KCC2 and pKCC2 in cultured hippocampal neurons (Leonzino et al. 2016). Taken together, the OXT‐OXTR‐pKCC2 pathway is critical to control the developmental GABAergic shift and thus the inhibition of nociceptive input in the dorsal spinal cord.

Given the evidence, we hypothesized that neonatal procedural pain leads to hypermethylation of *Oxtr* in the lumbar spinal cord, thereby preventing the developmental increase in KCC2 and pKCC2. Accordingly, this study aimed to investigate the effect of neonatal procedural pain on the expression of KCC2 and pKCC2 at the end of the developmental switch, i.e., P10 (Leonzino et al. 2016; Stil et al. 2009), and assessed needle prick‐induced changes in *Oxtr* methylation in the spinal cord at P10.

## 2 Materials and Methods

### 2.1 Ethics Statement

All animal experiments were performed in accordance with the European Directive for Protection of Vertebrate Animal Use for Experimental and Other Scientific Purposes (86/609/EEC) and were approved by the Committee for Experiments on Animals, Maastricht, the Netherlands (DEC 2017‐017).

### 2.2 Animals

For this study, 67 Sprague Dawley (SD) male and female rat pups from six time‐pregnant SD dams were used (Charles River). Breeding of dams was performed at the animal facilities of Maastricht University. On the day of birth, referred to as post‐natal day 0 (P0), the litters were culled to a maximum of N = 10, and pups were randomly assigned to neonatal conditions using an online randomization tool (random.org; Table 1). When litters exceeded 10 pups, additional pups were allocated to the P0 group and culled. All animals were housed in a room with controlled temperature (19–24°C) and humidity (55 ± 15%), a reversed 12 h/12 h day‐night cycle, and background music. Ad libitum water and food were available throughout the whole study period.

**TABLE 1:** Animals and sample distribution. At postnatal day 0 (P0) spinal cords were collected, or pups were assigned to neonatal condition: Needle prick (NP); disturbed control (DC) and undisturbed control (UC). While UC pups were left undisturbed, NP and DC pups underwent behavioral testing of paw withdrawal threshold (PWT). At P10 animals from all the litters were sacrificed and lumbar spinal cords were dissected. Animals were either assigned to the immunoblot quantification of pKCC2 and KCC2 protein levels or to the pyrosequencing *Oxtr* methylation levels analysis. One NP sample did not meet the quality control for pyrosequencing and was excluded from the statistical analysis. ♂: male; ♀: Female.

	PWT			pKCC2 and KCC2 protein quantification			*Oxtr* methylation levels		
	♂	♀	Total	♂	♀	Total	♂	♀	Total
NP	12	7	**19**	5	4	**9**	6	3	**9**
DC	12	8	**20**	5	4	**9**	7	4	**11**
UC	—	—	—	4	5	**9**	5	6	**11**
P0	—	—	—	4	4	**8**	—	—	—

### 2.3 Neonatal Procedures

To model repetitive procedural pain exposure in the NICU, the validated and reproducible needle prick model was used (Baudat et al. 2024; van den Hoogen et al. 2020, 2018; Knaepen et al. 2013; de Kort et al. 2021, 2022a). In brief, newborn pups were noxiously stimulated four times a day (8.00 am, 9.00 am, 10.00 am, 11.00 am) via unilateral 2 mm calibrated needle pricks in the mid‐plantar surface of the left hind paw from P0 to P7 (needle prick, NP, N = 19). Two control groups were used: disturbed controls (DC, N = 20) were pups disturbed for removal of the nest, short handling at the same hourly interval as NP animals, and von Frey testing, and then returning to the nest. The second control group were pups left undisturbed throughout the neonatal week (undisturbed control, UC, N = 20).

### 2.4 Von Frey for Mechanical Sensitivity in Neonates

Paw withdrawal thresholds (PWT) of the ipsi‐ and contralateral hind‐paws were assessed before (7.30 am; Baseline; BL) and 1 (12.00 am), 3 (2.00 pm), and 5 (4.00 pm) hours after the last noxious stimulation or handling using dorsal von Frey. Ascending Von Frey filaments (bending force 0.407 g, 0.692 g, 1.202 g, 2.041 g, 3.63 g from P4 onwards and 5.495 g from P6 onwards; Stoelting, Wood Dale, IL) were applied 5 times to the dorsal surface of the hind‐paws (Baudat et al. 2024; van den Hoogen et al. 2020, 2018; Knaepen et al. 2013; de Kort et al. 2021, 2022a). The number of positive responses, i.e., paw withdrawal or flinching behavior evoked by the filaments, was recorded per filament. Behavioral testing was discontinued when five positive responses were observed. A 50% PWT was calculated using a sigmoidal fitting curve in GraphPad Prism 9.5.1 (GraphPad Software, San Diego, CA).

### 2.5 Tissue Collection

Pups assigned to the P0 group were decapitated on P0 and dissected to collect the lumbar spinal cord (N = 8), followed by snap‐freezing in liquid nitrogen. Ipsi‐ and contra‐lateral sides were separated and stored at ‐80°C until further processing. At P10, all other animals (N = 59) were weighted and randomly, alternately collected from each nest and sacrificed. Animals were decapitated and dissected to collect the lumbar spinal cord, followed by snap‐freezing in liquid nitrogen. Only the lumbar part of the spinal cord was collected, and ipsi‐ and contra‐lateral sides were separated. All tissues were stored at ‐80°C until further processing.

### 2.6 DNA Isolation and Purification

DNA was extracted using a standard phenol/chloroform‐isoamyl alcohol (PCI) extraction method. In brief, the samples were lysated in 500 µL lysis buffer containing 50 mM Tris (pH 8.0), 1 mM EDTA, and 0.5% SDS. After adding 25 µL of proteinase K (Thermo Fisher Scientific, Waltham, MA), the samples were incubated overnight at 56°C in a shaking thermoblock. Following incubation, the proteinase K was inactivated at 80°C for 10 min. PCI (#77617‐100, Sigma Aldrich, Saint Louis, MO) was added in a 1:1 ratio, the samples were manually mixed for 5 min, and then centrifuged at 14.000 rpm for 5 min. The upper phase was carefully transferred to a new sterile 1.5 mL Eppendorf tube. Another equal volume (1:1 ratio) of PCI was added, after which the samples were mixed for 5 min and centrifuged at 14.000 rpm for 5 min. The upper phase from each sample was once again transferred to a new sterile 1.5 mL Eppendorf tube. The DNA was precipitated by adding 50 µL of 3 M NaAc (pH 5.6) and 1250 µL of 100% cold (‐20°C) ethanol, then incubated for at least 30 min at ‐80°C and centrifuged for 30 min at 14.000 rpm at 4°C. Subsequently, the solution was carefully removed, and the DNA pellets were washed using 70% cold ethanol and centrifuged for 5 min at 14.000 rpm at 4°C. Next, the ethanol was carefully removed, and the DNA pellets were air‐dried at room temperature. Finally, the isolated DNA from each sample was dissolved in 50 µL Milli‐Q and then stored at −20°C until further processing. The DNA yield of each sample was quantified by using the Qubit dsDNA HS Assay Kit (Invitrogen, Waltham, MA).

### 2.7 DNA Bisulfite Conversion

The EZ DNA Methylation‐Gold Kit (#D5008, Zymo Research, Irvine, CA) was used according to the manufacturer's instructions to bisulfite convert 400 ng of each DNA sample. Briefly, DNA was incubated with the CT conversion reagent for 8 min at 98°C, followed by a 150 min incubation at 64°C and a final storage step at 4°C. After the bisulfite clean‐up procedure, each sample was collected in a single 1.5 mL Eppendorf tube by flushing the spin column twice using 20 µL of elution buffer, resulting in a final concentration of 10 ng/µL for each fraction when assuming full recovery of the bisulfite‐converted DNA. Samples were randomized across bisulfite conversion plates and processed simultaneously to avoid batch effects. Bisulfite‐converted DNA was aliquoted and stored at ‐20°C until further processing.

### 2.8 Polymerase Chain Reaction

Polymerase chain reaction (PCR) primers and a sequencing primer targeting the *Oxtr* promotor were designed using the PyroMark Assay Design 2.0 software (Qiagen, Hilden, Germany) and were based on the Ensembl mRatBN7.2 genome build (Table 2). PCR amplification of the targeted region was performed with an initial denaturation step at 95°C for 5 min, followed by 55 cycles at 95°C, 56°C, and 72°C for 30, 30, and 30 s, respectively, with a final extension step at 72°C for 1 minute. For each PCR reaction, 10 ng of the bisulfite‐converted DNA was used. Each reaction contained 2.5 µL PCR buffer (10X) with 20 mM MgCl2, 0.5 µL 10 mM dNTP mix, 1 µL of each respective primer (5 µM stock), and 0.2 µL (5 U/µL) FastStart Taq DNA Polymerase (Roche Diagnostics GmbH, Mannheim, Germany) in a total volume of 25 µL. The PCR products were visualized on a 2% agarose gel. To concentrate the PCR product before pyrosequencing, 20 µL of reaction was speed vacuumed and resuspended in 10 µL of the DNA‐free H20.

**TABLE 2:** Primers used for bisulfite pyrosequencing of the Oxtr promotor in the spinal cord of P10 rats. CpG8 was excluded from the analysis because 8 samples did not meet the quality control standards of the Pyromark Q48 Autoprep software.

PCR primers				
Forward primer (5'‐3')	Reverse Primer (5'‐3')			Product size
GTTATTAGTAAAGTAGTAAGTTAGGGGATT	(Bio‐)ACAAATTAACCCAA AATTACCTTACC			174
**Pyrosequencing primers**				
Sequencing primer (5'‐3')		Number of CpG sites	Genomic coordinates (Ensembl, mRatBN7.2)	
AGTAGTAAGTTAGGGGATTTTAA		8	4:145615606‐145615724	

### 2.9 Pyrosequencing

The Pyromark Q48 Autoprep system with the PyroMark Q48 Advanced CpG Reagents (Qiagen) and PyroMark Q48 Magnetic Beads (Qiagen) was used for bisulfite pyrosequencing according to the manufacturer's instructions. All pyrosequencing assays were tested for their sensitivity on various fractions, i.e., 0%, 25%, 50%, 75%, and 100% of methylated DNA standards. Modification levels at a single CpG resolution were analyzed with the Pyromark Q48 Autoprep software (Qiagen).

### 2.10 Western Blot

Spinal cord samples were manually homogenized and lysed in 50 µL of RIPA buffer (Thermo Fisher) containing phosphatase inhibitor (Sigma) and protease inhibitor (Sigma). Homogenized tissues were centrifuged at max speed (15’000xg) for 15 min at 4°C, and the supernatant was transferred to a new clean Eppendorf tube. Protein quantification was performed using the detergent‐compatible (DC) Protein Assay kit (BioRad, Hercules, CA, USA). Homogenized tissues were kept on ice in between steps and stored at ‐80°C in‐between processing.

For the western blot, proteins were denatured for 10 min at 70°C using the lithium dodecyl sulfate (LSD) sample buffer. For each sample, 20ug of protein was separated using the 10% SDS‐polyacrylamide gel (BioRad), followed by a transfer on a nitrocellulose membrane using the semi‐dry blot transfer system (BioRad). Blots were incubated for 1 h with Odyssey blocking buffer (Li‐Cor, Lincoln, NE, USA). For KCC2 quantification, blots were incubated overnight at 4°C with rabbit anti‐KCC2 antibody (1:1000, Sigma, lot#3872327) and mouse anti‐GAPDH (1:1,000,0000, Thermo Fisher), diluted in 1:1 PBS:Odyssey blocking buffer (Li‐Cor). The next day, blots were washed for 3 × 5 min with PBS containing 0.01% Tween (PBS‐T) followed by 1‐hour incubation with secondary antibodies, including goat anti‐rabbit IRDye 800 (1:10,000, Li‐Cor) and donkey anti‐mouse IRDye 600 (1:10,000, Li‐Cor). Membranes were subsequently washed for 3 × 5 min with PBS‐T.

For pKCC2 quantification, blots were washed for 3 × 5 min in TBS following blocking using the Odyssey blocking buffer. Then blots were incubated for 30 min with streptavidin (0.05 mg/ml, Thermo Fisher) diluted in TBS containing 0.01% of Tween (TBS‐T) followed by 3 × 5 min of washing in TBS‐T. Blots were then incubated for 30 min with 0.25 mg/ml of biotin (Sigma) diluted in TBS‐T and subsequently washed for 3 × 5 min in TBS‐T. Blots were incubated overnight at 4°C with a rabbit anti‐pKCC2 antibody (1:750, Thermo Fisher, lot#YC3869592) and a mouse anti‐GAPDH (1:1,000,0000, Thermo Fisher), diluted 1:1 in TBS:Odyssey blocking buffer (Li‐Cor). The next day, blots were washed for 3 × 5 min with TBS‐T followed by a 1‐hour incubation with secondary antibodies, including biotinylated goat anti‐rabbit (1:500, Li‐Cor) and biotinylated donkey anti‐mouse (1:500, Li‐Cor). Blots were then washed for 3 × 5 min in TBS‐T and incubated with dye (Li‐Cor) for one hour. Finally, blots were washed for 3 × 5 min in TBS‐T. For both KCC2 and pKCC2, all steps were performed at room temperature and in the dark unless stated otherwise.

All membranes were scanned and visualized using the Odyssey CLx Infrared Imaging System (Li‐Cor). Protein band quantification was performed using the built‐in program of ImageStudio Lite (version 5.2, Li‐Cor). KCC2 and pKCC2 raw intensities were normalized to GAPDH for loading differences and adjusted to a control sample present on all blots. The control sample was a homogenate of randomly selected sample lysates. Conditions were distributed across gels, and the experimenter was blinded to neonatal conditions.

### 2.11 Statistics

For all data, the normality assumption was evaluated using Q‐Q plots and the Shapiro‐Wilk normality test. The presence of outliers was assessed using the extreme value analysis built‐in SPSS software version 28.0.1.1. Differences in weight and mechanical sensitivity during the neonatal period were analyzed using a repeated measure analysis of variance (ANOVA; assessing the effects of age and condition) followed by pairwise comparisons with Sidak correction for multiple testing. Sex was included as a covariate. KCC2, pKCC2, and pKCC2/KCC2 levels were analyzed using a non‐parametric Kruskal‐Wallis test, and pairwise comparisons were corrected with Bonferroni correction. All analyses were performed on SPSS version 28.0.1.1., and results were considered significant at p < 0.050.

Average methylation levels were analyzed using a mixed ANOVA model using SPSS software version 28.0.1.1. Neonatal conditions were defined as a between‐subject variable, side (ipsi‐ versus contra‐lateral) was considered a within‐subject variable, and sex was added as a covariate. When a significant effect for the between‐ or the within‐subject variable was obtained, subsequent t‐tests were performed for relevant comparisons only. The average methylation level (Ensembl, mRatBN7.2, 4:145615606‐145615724) was calculated by averaging the methylation levels of all cytosine‐phosphate‐guanine (CpG) sites. Methylation differences were also analyzed for each CpG site using a repeated measure mixed‐ANOVA, with between and within‐subject variables as described above, and CpGs as repeated measures. CpG site 8 was excluded from analysis due to not meeting the quality control standards of the Pyromark Q48 Autoprep software for 8 out of 31 samples. One sample was excluded from all analyses after not meeting the quality control standards of the Pyromark Q48 Autoprep software on all CpGs. Results were considered significant at p < 0.05. All data was plotted using GraphPad Prism 10.

## 3 Results

### 3.1 Neonatal Needle Pricks, but Not Handling, Increase Mechanical Sensitivity in the Ipsilateral Paw

**FIGURE 2:**
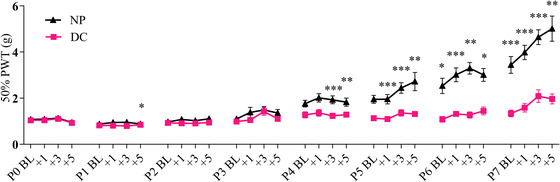
Ipsilateral mechanical sensitivity after needle prick or handling during the neonatal week. From post‐natal day 5 (P5), repetitive needle prick (NP, N = 19) results in decreased ipsilateral paw withdrawal threshold (PWT) compared to handling (DC, N = 20; F_1, 36_ = 29.508, p < 0.001). An effect not observed on the contralateral hind paw (F_1, 36_ = 0.028, p = 0.867). From P0 to P7, ipsilateral PWT significantly increased over time independently of neonatal condition. Postnatal day 0 to 7 (P0‐7); BL, baseline von Frey measurement; +1/+3/+5, von Frey measurement 1/3/5 h after last needle pricks on P0‐P7. Data plotted as mean ± standard error of the mean (SEM). * p < 0.05; ** p < 0.01; *** p < 0.001.

In line with previous studies, no effect of sex was observed on the ipsilateral (F_1, 36_ = 0.348, p = 0.559) and contralateral (F_1, 36_ = 0.723, p = 0.401) PWT (Baudat et al. 2024; van den Hoogen et al. 2020, 2018; de Kort et al. 2021). Ipsilateral (F_31, 1116_ = 3.827, p < 0.001; Figure 2) and contralateral (F_31, 1116_ = 8.207, p < 0.001) PWT significantly increased over time. The interaction of time and neonatal condition was significant only on the ipsilateral side (F_31, 1116_ = 17.283, p < 0.001), and a main effect of neonatal condition was observed on the ipsilateral PWT (F_1, 36_ = 29.508, p < 0.001; Figure 2). Pairwise comparisons revealed that NP animals had significantly lower PWT at P2 + 3 h; and from P4 + BL onwards when compared to DC animals. Contralateral PWT was not affected by the neonatal condition (F_1, 36_ = 0.028, p = 0.867). Animals weight significantly and consistently increased over the post‐natal week (F_7, 252_ = 50.403, p < 0.001) and was not affected by neonatal condition (F_1, 36_ = 2.861, p = 0.099). Sex significantly affected weight intake (F_1, 36_ = 8.762, p = 0.005). The increase in mechanical hypersensitivity observed throughout the neonatal week confirms that animals developed neonatal procedural pain (Baudat et al. 2024; van den Hoogen et al. 2020, 2018; Knaepen et al. 2013; de Kort et al. 2021).

### 3.2 Neonatal Needle Pricks Reduce the Phosphorylation of KCC2 at the ser940 Residue in the Ipsilateral Spinal Cord

**FIGURE 3:**
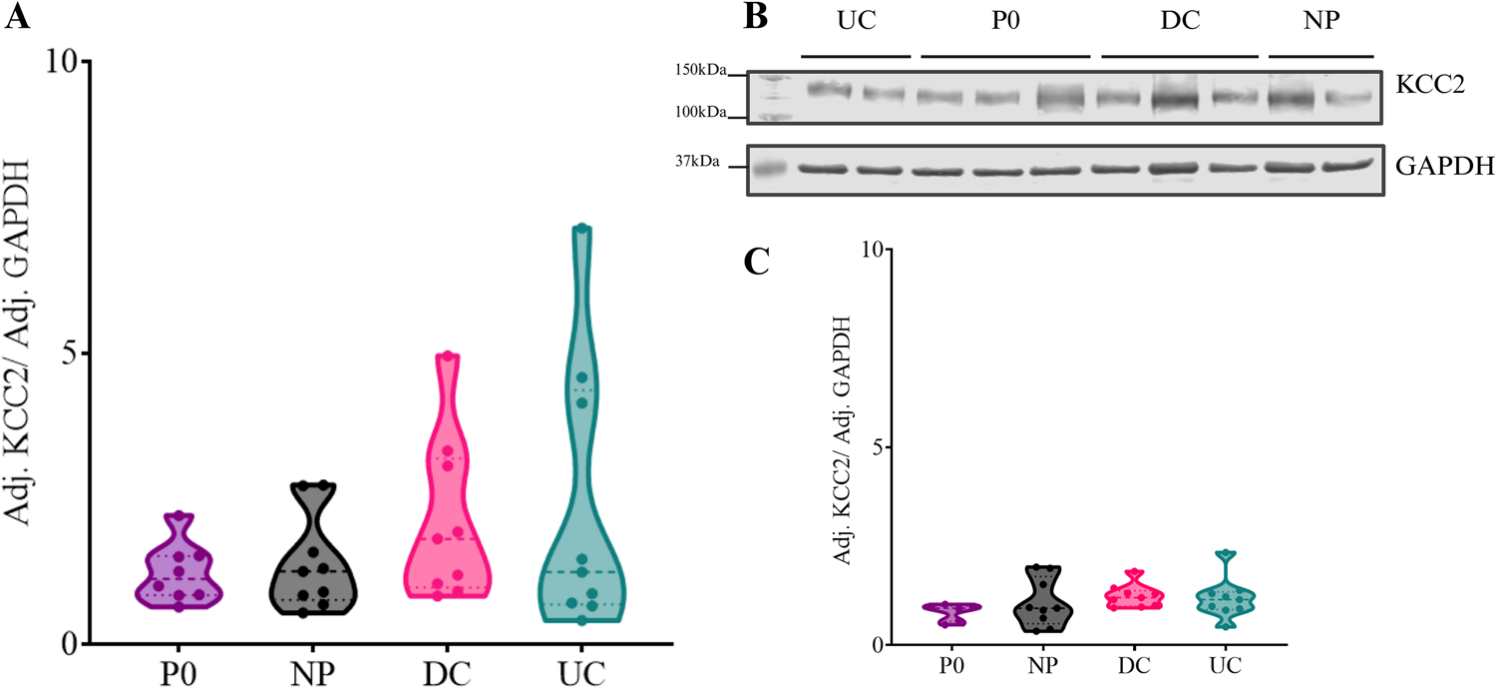
Neonatal needle pricks do not alter lumbar spinal KCC2 protein levels. (A) No effects of neonatal conditions were observed on ipsilateral lumbar spinal KCC2 levels at P10 (H_3_ = 1.924, p = 0.588). (B) Immunoblot of lumbar spinal KCC2 normalized over GAPDH. (C) No effects of neonatal conditions were observed on contralateral lumbar spinal KCC2 levels at P10 (H_3_ = 5.037, p = 0.169). Needle prick (NP, N = 9); Disturbed control (DC, N = 9); Undisturbed control (UC, N = 9); P0 naïve animals (P0, N = 5). Two P0 samples were excluded due to abnormal protein separation. Data plotted as median ± quartile lines. Created with BioRender.com.

**FIGURE 4:**
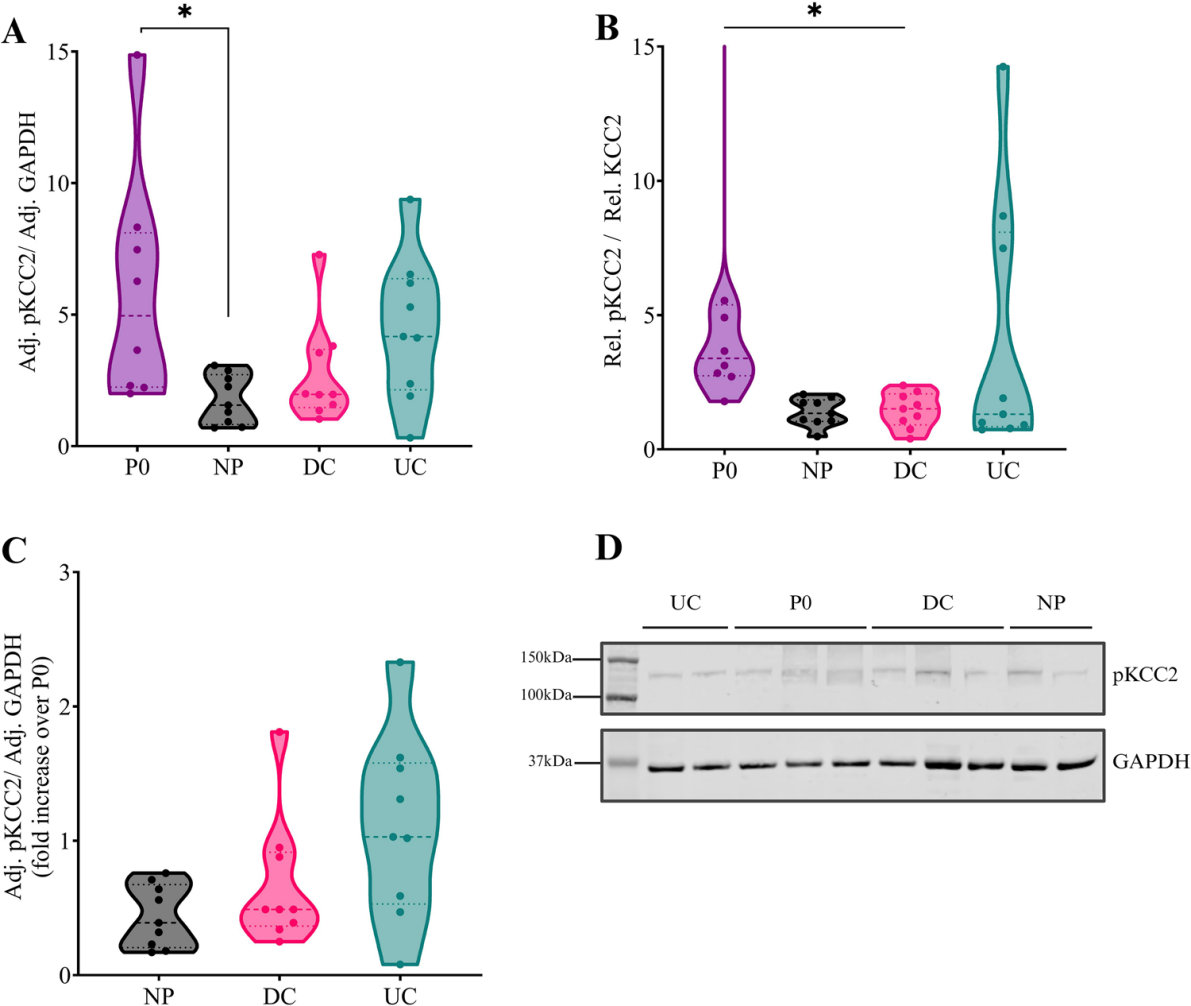
Neonatal needle pricks alter the developmental decrease in ipsilateral lumbar spinal pKCC2 protein levels. (A) Neonatal needle pricks (NP, N = 9) decrease the phosphorylation of KCC2 ser940 residue (pKCC2) in the ipsilateral spinal cord of post‐natal day 10 (P10) animals compared to P0 naïve animals (N = 7) (H_3_ = 9.950, p = 0.019). This effect was not observed in the contralateral lumbar spinal cord (H_3_ = 1.811, p = 0.613). (B) Animals from the NP and handling (DC, N = 9) groups displayed a lower ipsilateral spinal pKCC2/KCC2 ratio at P10 compared to P0 naïve animals (H_3_ = 10.658, p = 0.014). (C) Ipsilateral pKCC2 levels were normalized over P0 levels, and NP animals showed a trend towards a significant developmental decrease in pKCC2 levels as compared to the undisturbed animals (UC, N = 9; p = 0.053). (D) Immunoblot of lumbar spinal pKCC2, normalized over GAPDH. Adj.: adjusted to control protein sample. Rel.: normalized and relative to Adj. GAPDH (e.g.: Rel. KCC2 = Adj.KCC2 / Adj.GAPDH). Data plotted as median ± quartile lines. *p < 0.05. Created with BioRender.com.

In the ipsilateral spinal cord, neonatal conditions did not alter the KCC2 protein levels (H_3_ = 1.924, p = 0.588, Figures 3A, 3B, and 3C), but did affect pKCC2 protein levels (H_3_ = 9.950, p = 0.019). Pairwise comparisons showed lower ipsilateral pKCC2 protein levels in NP animals as compared to P0 animals (H_1_ = ‐13.750, p = 0.035), an effect that was not observed in other neonatal (DC or UC) conditions (Figures 4A and 4D). The ratio of pKCC2 over KCC2 was significantly affected by neonatal condition on the ipsilateral side (H_3_ = 10.658, p = 0.014), and the pairwise comparisons identified lower ratios of pKCC2/KCC2 in the NP (p = 0.022) and the DC (p = 0.034) animals compared to the P0 naïve animals at P10 (Figure 4B). Once normalized over P0, a trend towards significance for the neonatal condition was identified for the pKCC2 (H_2_ = 5.686, p = 0.058, Figure 4C) levels but not the KCC2 (H_2_ = 1.316, p = 0.518) or the pKCC2/KCC2 ratio (H_2_ = 0.088, p = 0.957). Pairwise comparisons highlighted that the NP, but not the DC animals, displayed lower pKCC2 levels as compared to UC animals (p = 0.053). On the other hand, the neonatal conditions did not alter the contralateral lumbar spinal levels of KCC2 (H_3_ = 5.037, p = 0.169), pKCC2 (H_3_ = 1.811, p = 0.613), nor the ratio of pKCC2/KCC2 (H_3_ = 3.97651, p = 0.2). After normalization over P0, no significant effects of neonatal conditions were observed on the contralateral side for pKCC2 (H_2_ = 1.961, p = 0.375), and KCC2 (H_2_ = 1.633, p = 0.442) protein levels, nor for the pKCC2/KCC2 ratio (H_2_ = 3.623, p = 0.163). Together, those results indicate that neonatal procedural pain decreases spinal pKCC2 levels compared to P0 levels.

### 3.3 Neonatal Procedural Pain Does Not Change the Methylation Status of *Oxtr* Promotor in the Spinal Cord

**FIGURE 5:**
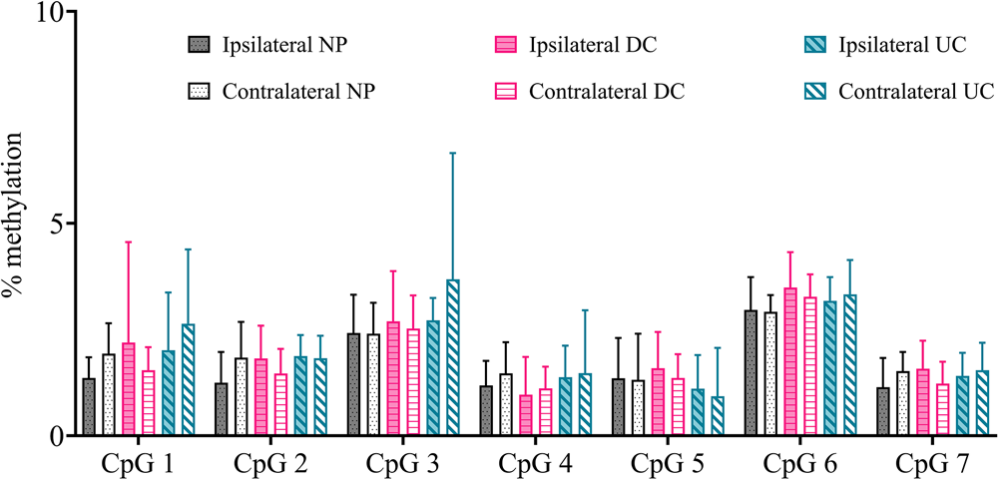
Neonatal needle pricks do not alter the methylation status of the *Oxtr* promotor in the spinal cord of P10 animals. Methylation levels significantly varied across CpG sites (F_1, 150_ = 8,130, p < 0.001). Needle pricking (NP, N = 9), handling (DC, N = 11) or being undisturbed (UC, N = 9) did not significantly affect methylation levels of the CpG sites (F_2, 25_ = 1.084, p = 0.513) or the average methylation percentage over the targeted region (F_1, 25_ = 0.689, p = 0.511). Ipsilateral and contralateral methylation levels do not significantly differ (F_1, 150_ = 1,405, p = 0.247). Data plotted as mean ± standard error of mean (SEM).

At P10, methylation levels significantly varied depending on the CpG site (F_1, 150_ = 8,130, p < 0.001) but were not significantly affected by side (ipsilateral versus contralateral, F_1,150_ = 1,405, p = 0.247), neonatal conditions (F_2, 25_ = 1.084, p = 0.513) or sex (F_1, 25_ = 3.226, p = 0.085; Figure 5). No significant interaction effect between side and sex (F_1, 25_ = 1.645, p = 0.211), side and neonatal condition (F_2, 25_ = 2.559, p = 0.097), or CpG site and neonatal conditions (F_12, 150_ = 0.952, p = 0.123) was observed. Average methylation levels were also not significantly affected by side (F_1, 25_ = 1.463, p = 0.238), neonatal conditions (F_1, 25_ = 0.689, p = 0.511) or sex (F_1, 25_ = 3.197, p = 0.086). No significant interaction effect between side and sex (F_1, 25_ = 1.714, p = 0.202), or side and neonatal condition (F_2, 25_ = 2.582, p = 0.096) were observed. Collectively, these results demonstrate that neonatal procedural pain does not affect methylation of the studied *Oxtr* promotor region at P10.

## 4 Discussion

The present study investigated the effect of neonatal procedural pain on the GABAergic shift in the lumbar spinal cord of P10 rat pups, using the needle prick model. More specifically, changes in the developmental expression of KCC2 and pKCC2, as well as alterations in the *Oxtr* promotor methylation, were assessed in P10 animals, once the GABAergic shift was completed (Leonzino et al. 2016; Stil et al. 2009). As hypothesized, neonatal procedural pain resulted in reduced pKCC2 levels in the ipsilateral spinal cord, compared to P0 naïve animals, an effect that was not observed in other neonatal conditions or the contralateral side. Given the role of Ser940 phosphorylation in the membrane stability and activity of KCC2 (Lee et al. 2007), our results suggest a reduced spinal KCC2 function at P10. Consequently, needle pricks‐induced pKCC2 downregulation may drive a higher intracellular Cl^−^ concentration, hindering the GABAergic shift and the excitatory versus inhibitory balance (Figure 1C). This aligns with previous studies showing disruption of the excitatory versus inhibitory balance in the spinal nociceptive network of adult rats exposed to neonatal pain (van den Hoogen et al. 2018; Li et al. 2015; Li and Baccei 2014, 2016). Although research has yet to show the effect of neonatal procedural pain on adult pKCC2, we hypothesize that pKCC2 downregulation after neonatal procedural pain lasts into adulthood and participates in the alteration of adult spinal excitatory versus inhibitory balance. To specifically elucidate the impact of neonatal procedural pain on adult GABAergic inhibition and spinal excitability, future research should extend this investigation to adult spinal pKCC2 expression patterns. Altogether, the findings demonstrate an alteration of pKCC2 in response to neonatal procedural pain (Figure 1C), which could delay or prevent the GABAergic shift, and contribute to the long‐term alterations observed in adult nociceptive behavior.

A possible explanation for the impairment of pKCC2 stems from the interaction between the NMDA receptor and KCC2 (Hori and Kanda 1994; Lee et al. 2011). Repetitive nociceptive stimulation of post‐synaptic neurons recruits NMDA receptors, which play a key role in the process of central sensitization (Basbaum et al. 2009). This phenomenon involves the heightened responsiveness of post‐synaptic neurons, leading to an amplified transmission of noxious signals to supra‐spinal areas. An interaction between NMDA and KCC2 phosphorylation exists, as highlighted by the recruitment of protein‐phosphatase 1 (PP1) (Lee et al. 2011). The latter participates in the de‐phosphorylation of pKCC2 and reduces KCC2 presence at the cell surface upon NMDA‐dependent calcium (Ca^2+^) influx (Lee et al. 2011). Lee and colleagues further demonstrated that glutamate exposure shifts the GABA response to depolarizing and thus excitatory neurotransmission in cultured rat hippocampal neurons (Lee et al. 2011). Given the role of NMDA in the sensitization of post‐synaptic neurons, NDMA‐induced Ca^2+^ influx may participate in the de‐phosphorylation of pKCC2 observed in the needle prick model.

A second aspect of the study was based on the hypothesis that neonatal procedural pain modifies the methylation pattern of the *Oxtr* promotor in P10 spinal cords, participating in the disruption of the GABAergic shift. Our results showed no effects of neonatal procedural pain on spinal methylation patterns of the assessed *Oxtr* promotor region. Methylation patterns of the assessed *Oxtr* region were shown to be sensitive to early life events, as lower methylation was observed in the same promotor region in the plasma of adult rats with a history of low maternal care (Beery et al. 2016). Note that Berry and colleagues defined low maternal care based on maternal licking frequency and did not include stimulation of the nociceptive circuits in their study. The same research highlighted a tissue‐dependent effect of maternal care on *Oxtr* methylation (Beery et al. 2016), implying that methylation changes following neonatal procedural pain may still occur outside of the lumbar spinal cord or only within specific cell types within the lumbar spinal cord. It should also be noted that changes in *Oxtr* methylation patterns are not limited to the CpG island assessed in our study. Indeed, *Oxtr* is composed of multiple exons and introns, among which methylation patterns of exon III (hg38, 3:8767589‐8768307) are susceptible to early‐life adverse events (Kraaijenvanger et al. 2019). Additionally, a clinical study observed an upregulation of plasma *OXTR* exon III methylation levels 10 min after the trier social stress test and a return to baseline 90 min after the stress test (Unternaehrer et al. 2016). This previous research, though requiring cautious interpretation due to the small methylation changes and lack of replication, highlights that stress‐dependent methylation changes to OXTR promotor are reversible within 90 min (Unternaehrer et al. 2016). In our experiments, spinal methylation levels were only measured at P10, limiting our ability to detect acute and reversible changes to the *Oxtr* promotor methylation. Furthermore, other epigenetic mechanisms, such as histone acetylation, might participate in the regulation of *Oxtr* by neonatal procedural pain. In a mice paradigm of maternal experience, histone acetylation of *Oxtr* contributes to maternal memory (Stolzenberg et al. 2014), and maternal obesity increases histone acetylation at the *Oxtr* transcription starting site in male, but not female mice offspring, a finding consistent with OXTR mRNA upregulation (Glendining and Jasoni 2019). Taken together, further studies on the epigenetic regulation of *Oxtr* are essential to fully grasp the effects of neonatal procedural pain and its role in the OT‐OXTR‐pKCC2 pathway of the GABAergic shift.

Despite the impact of our findings, it is important to address several limitations. First, a lower pKCC2/KCC2 ratio was detected in needle‐pricked and handled animals on the ipsilateral side, and no significant developmental increase in KCC2 protein levels in the rat lumbar spinal cord was observed. The reduced pKCC2/KCC2 ratio was not seen in the contralateral spinal cord of DC animals, which do not receive unilateral treatment. In contrast to our findings, other studies have reported a gradual upregulation of KCC2 protein expression in the healthy rat lumbar spinal cord between P0 and P20 (Gazzo et al. 2021; Stil et al. 2009). Leonzino and colleagues also described an upregulation of KCC2, pKCC2, and pKCC2/KCC2 between P0 and P6 in the mouse hippocampus and primary cultured hippocampal cells (Leonzino et al. 2016). In both studies, animals were undisturbed throughout the neonatal week, modeling the UC group. Our observation that KCC2 protein levels did not increase throughout development may stem from the limitations inherent to western blot analysis. This method, while useful for relative protein comparison, is often accompanied by variability due to factors like antibody specificity, signal saturation, and transfer efficiency. A second limitation is the absence of electrophysiological data and intracellular Cl^−^ concentration measurements, leaving room for further investigation into the functional implications of needle prick‐induced pKCC2 downregulation. Next, other post‐translational mechanisms must be taken into consideration to establish KCC2 activity and the state of the GABAergic shift. For instance, in developing mice, de‐phosphorylation of the KCC2 threonine residues 906 and 1007 (Thr906/Thr1007) increased as the GABAergic transmission shifts from excitatory to inhibitory and enhanced KCC2 activity by more than ten‐fold (Watanabe et al. 2019). To unequivocally determine the effects of neonatal procedural pain on the GABAergic shift, future studies should provide a more comprehensive view, including Thr906/Thr1007 phosphorylation, intracellular Cl^−^ concentration, and GABA‐evoked cellular depolarization in the developing lumbar spinal cord.

Treating neonatal procedural pain with adequate analgesics prevents the emergence of long‐term behavioral effects associated with a disruption of the excitatory versus inhibitory balance, such as longer post‐operative pain (van den Hoogen et al. 2018, 2016, 2021, 2019; de Kort et al. 2022b). Given the disruption of the GABAergic shift and the long‐lasting alteration of the excitatory versus inhibitory balance in the needle prick model, pharmacological treatment targeting the GABA system is of interest to the NICU. For instance, activation of the GABA_A_ receptor in cultured hippocampal neurons upregulates KCC2 and promotes the GABAergic shift and thus may support the completion of the developmental GABAergic shift in the spinal cord (Ganguly et al. 2001). Similarly, the use of the GABA analog, Gabapentin, indirectly modulates KCC2 activity and was shown to alleviate pain (Eroglu et al. 2009; Huang et al. 2024; Taylor et al. 1998). To this day, the treatment with Gabapentin in the NICU for pain management is limited but was shown to decrease pain scores and reduce the need for additional analgesics (Burnsed et al. 2020; Sacha et al. 2017). The use of preclinical models, such as the needle prick model, can provide evidence‐based insights into Gabapentin's safety and effectiveness in managing neonatal procedural pain in the NICU.

In conclusion, neonatal procedural pain affects the phosphorylation of KCC2 at the Ser940 residue in the lumbar spinal cord of P10 rats, possibly leading to a compromised GABAergic shift and a lasting excitatory versus inhibitory imbalance in the spinal cord.

## Author Contributions

All authors substantially contributed to the conception and design of the experiment, interpreted the data and critically revised the manuscript. M. Baudat performed the acquisition of data and drafting of the article. The final manuscript was approved by all authors.

## Conflicts of Interest

The authors declare no conflict of interest.

## Ethics Statement

All animal experiments were performed in accordance with the European Directive for Protection of Vertebrate Animal Use for Experimental and Other Scientific Purposes (86/609/EEC) and were approved by the Committee for Experiments on Animals, Maastricht, The Netherlands (DEC 2017‐017).

## Data Availability

The data that support the findings of this study are available from the corresponding author upon reasonable request.
